# A novel substitution in NS5A enhances the resistance of hepatitis C virus genotype 3 to daclatasvir

**DOI:** 10.1099/jgv.0.001496

**Published:** 2020-11-03

**Authors:** Guilherme Rodrigues Fernandes Campos, Joseph Ward, Shucheng Chen, Cintia Bittar, João Paulo Vilela Rodrigues, Ana de Lourdes Candolo Martinelli, Fernanda Fernandes Souza, Leonardo Régis Leira Pereira, Paula Rahal, Mark Harris

**Affiliations:** ^1^​ São Paulo State University, Institute of Biosciences, Languages and Exact Sciences, São José do Rio Preto, São Paulo State 15054-000, Brazil; ^2^​ School of Molecular and Cellular Biology, Faculty of Biological Sciences, and Astbury Centre for Structural Molecular Biology, University of Leeds, Leeds, LS2 9JT, UK; ^3^​ University of São Paulo, Ribeirão Preto School of Medicine, Ribeirão Preto, SP 14049-900, Brazil; ^4^​ University of São Paulo, Ribeirão Preto Faculty of Pharmaceutical Sciences, Ribeirão Preto, SP 14040-903, Brazil

**Keywords:** hepatitis C virus, genotype 3, NS5A, DAA resistance

## Abstract

Hepatitis C virus (HCV) genotype 3 presents a high level of both baseline and acquired resistance to direct-acting antivirals (DAAs), particularly those targeting the NS5A protein. To understand this resistance we studied a cohort of Brazilian patients treated with the NS5A DAA, daclatasvir and the nucleoside analogue, sofosbuvir. We observed a novel substitution at NS5A amino acid residue 98 [serine to glycine (S98G)] in patients who relapsed post-treatment. The effect of this substitution on both replication fitness and resistance to DAAs was evaluated using two genotype 3 subgenomic replicons. S98G had a modest effect on replication, but in combination with the previously characterized resistance-associated substitution (RAS), Y93H, resulted in a significant increase in daclatasvir resistance. This result suggests that combinations of substitutions may drive a high level of DAA resistance and provide some clues to the mechanism of action of the NS5A-targeting DAAs.

## Introduction

Hepatitis C virus (HCV) currently infects an estimated total of 70 million individuals worldwide. In 85 % of cases infection leads to chronic liver disease: fibrosis, cirrhosis and hepatocellular carcinoma (HCC). HCV has a positive-strand RNA genome and is the most variable human virus, classified into seven genotypes (GTs), exhibiting >30 % nucleotide sequence divergence, with each GT being further divided into subtypes [1]. GT3 is the second most common GT (after GT1) and accounts for 30 % of global HCV cases [2, 3]. The variability of the virus results from a lack of proofreading activity of the viral RNA-dependent RNA polymerase (NS5B) and importantly enables the emergence of mutations that can lead to evasion of both the immune response and antiviral treatments.

The development of direct-acting antivirals (DAAs) has revolutionized the treatment of HCV infection. In Brazil, since 2015, the administration of pegylated interferon-α and ribavirin has only occurred in a few cases, and the first generation of DAAs (telaprevir and boceprevir) has been discontinued. A second generation of DAAs is currently in use and comprises simeprevir (NS3 protease inhibitor), daclatasvir (NS5A inhibitor) and sofosbuvir (NS5B inhibitor). However, despite the high efficiency of this therapy, resistance is still reported in approximately 10 % of the patients, depending on the viral genotype and the disease progression level. Resistance is invariably linked to the presence of resistance-associated substitutions (RASs). Generally, these substitutions can be found easily and in high frequency after treatment failure, but in some cases they can be detected pre-treatment and rapidly increase in frequency due to the selective pressure of DAAs. Since the circulation of these pre-existing substitutions can impact on the efficacy of antiviral treatment, studying the phenotype of these substitutions with regard to DAA resistance and viral fitness is important to understand DAA failure.

Among all HCV genotypes, GT3 exhibits the greatest resistance to DAA therapy, with lower response rates compared to other genotypes. This response is even lower in patients with advanced liver disease, such as cirrhosis [3]. GT3 is the second most prevalent genotype in Brazil, being responsible for approximately 24 % of the infections, and is thus an important public health problem [4]. In this regard, this study focused on resistance to the NS5A-targeting DAA daclatasvir (DCV) with a view to characterizing the substitutions associated with treatment failure in GT3-infected patients. We observed a novel substitution in NS5A, serine 98 to glycine (S98G), that was present at high frequency in samples from relapsed patients compared to those who exhibited a sustained virological response (SVR). We characterized the phenotype of this substitution in the context of both replication fitness and resistance to DAAs, using two subgenomic replicon (SGR) systems recently developed for GT3.

## Methods

### Clinical samples

The population in this study consists a cohort of HCV GT3-infected patients under treatment with the NS5A inhibitor daclatasvir (DCV) and the NS5B inhibitor sofosbuvir (SOF). Serum samples from these patients were collected at Ribeirão Preto Medical School, University of São Paulo, Brazil. The samples were collected at two different time points, before and after treatment, where treatment response was assessed. Individuals who did not present an SVR after treatment were considered to be non-responders, while those in whom viral load was detected 24 weeks after treatment end were considered relapsers. Individuals whose viral load was not detected during treatment and at this last time point were classified as responders.

### Molecular analysis

RNA was extracted from serum using TRIzol according to the manufacturer’s instructions (Thermo Fisher Scientific), followed by cDNA synthesis using the High-Capacity cDNA Reverse Transcription kit (Applied Biosystems). Considering that DCV interaction with NS5A occurs in domain I, likely between amino acids 28 and 93 [5], we amplified and sequenced the first 300 nucleotides of the NS5A coding region. Amplification of the target region was performed by nested PCR using Long PCR Enzyme Mix (Thermo Fisher Scientific) and specific primers (sequences available upon request). Sequencing was performed using the Sanger method with BigDye Terminator v.3.1 (Applied Biosystems) using the same primers as the second PCR. Readings were performed in a 3130xl Genetic Analyzer (Applied Biosystems).

### Cell culture

Huh7.5 cells, a human hepatocellular carcinoma cell line, were cultured in Dulbecco’s modified Eagle’s medium (DMEM) supplemented with 10 % foetal bovine serum (FBS), 100 IU ml^−1^ penicillin and 100 µg ml^−1^ streptomycin. Cells were incubated at 37 °C and 5 % CO_2_.

### SGR constructs

The *in vitro* approaches in this study were based on two GT3a SGRs from different isolates, SGR-luc-S52 [6], derived from S52/SG-neo [7], and SGR-luc-DBN3a [8], derived from the DBN3a infectious clone [9]. As a negative control, we used the replication defective constructs with inactivating mutations in the RNA-dependent RNA polymerase, NS5B: GDD→GNN. This, and the other mutations, were introduced using the Q5 Site-Directed Mutagenesis kit (New England Biosciences).

### SGR replication assays

SGR RNA was produced by *in vitro* transcription and electroporated in Huh7.5 cells with the Gene Pulser Xcell Electroporation System (Bio-Rad), using the square wave protocol (260V, 25 ms, 1 pulse and 4 mm cuvette). For each electroporation, 2×10^6^ cells were added to the cuvette and, after the procedure, 10^4^ cells were seeded in each well, in a 96-well white plate. Cells were lysed using Passive Lysis Buffer (PLB) (Promega) and replication was analysed by measuring luciferase activity using the Luciferase Assay System kit (Promega). The assay was analysed at 24, 72 and 96 h post-electroporation (p.e.) for S52, and 24, 48 and 72 h p.e. for DBN3a. This difference in incubation period is due to the more efficient replication of DBN3a [9]. For the DAA assays, at 4 h p.e. DAAs were added to the media at the appropriate concentration ranges and incubated for 96 (S52) or 72 h (DBN3a). All assays were performed in triplicate and three independent assays were performed.

### Statistical analysis

Data were analysed using GraphPad Prism 5 software. The mean and standard deviation were determined, and all statistical analyses were performed using the analysis of variance (ANOVA) test and Dunnett’s multiple comparison test, considering *P*<0.05 as significant.

## Results

### Identification of a novel substitution in NS5A from patients who relapsed after DAA therapy

**Fig. 1.**
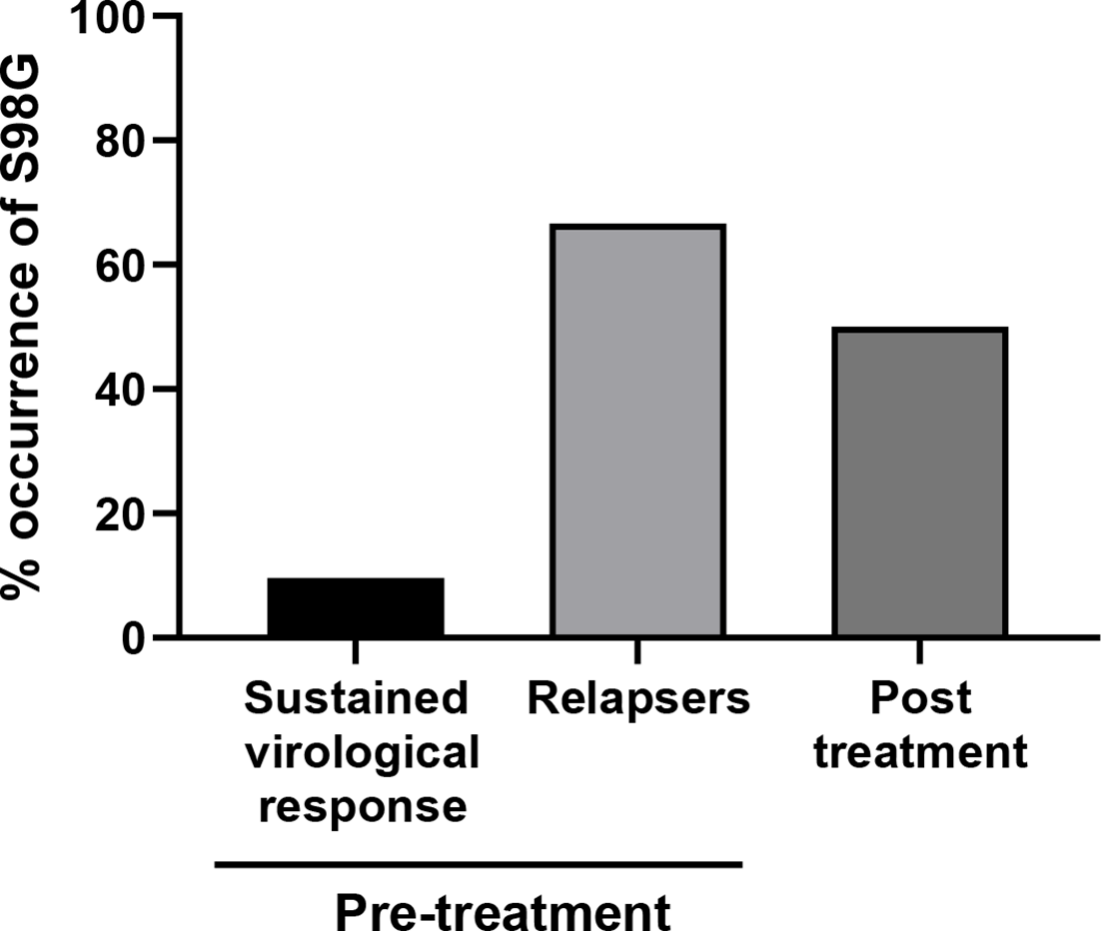
Frequency of S98G in the studied population.

This study analysed a cohort of 82 patients infected with HCV genotype 3 who were treated with a combination of sofosbuvir (SOF: NS5B inhibitor) and daclatasvir (DCV: NS5A inhibitor) for a period of 12 weeks. Serum samples from these patients were collected at Ribeirão Preto Medical School, University of São Paulo (USP), Brazil. The SVR rate for this cohort was 92.7 %. Although all of the patients responded to therapy, six of them then relapsed after the end of treatment. In an attempt to understand the molecular basis for relapse, we performed Sanger sequence analysis of NS5A domain I (corresponding to amino acids 28–100) both pre- and post-treatment. We detected a number of substitutions within the NS5A domain I coding sequence when compared to the consensus genotype 3 (NZL1) (see Table 1), but the most prevalent was S98G, which was present in 14.45 % of all pre-treatment samples. What was more interesting, however, was that this substitution was present in 66.6 % of the patients who subsequently relapsed, compared to 9.63 % of responders (Fig. 1). In the post-treatment samples, S98G was present in the relapsed patients at a frequency of 50 % (Table 2, Fig. 1). A comparison with 2915 genotype 3 isolates from different geographical regions showed that S98G is present at a frequency of 14.5 %, corresponding with our pre-treatment frequency. Of note, as expected, other previously described substitutions (A30K, A62T and Y93H) were also present at higher frequency in the post-treatment samples from the six relapsed patients (Table 2), but we focused on S98G, as it had not previously been associated with DAA resistance. We were intrigued that the presence of S98G in pre-treatment samples seemed to correlate with relapse after DCV treatment and we therefore set out to analyse the effect of this substitution on viral fitness and DAA sensitivity *in vitro*.

**Table 1.** Substitutions identified in this study; those in bold were previously unidentified

Substitution	Pre-treatment frequency	Post-treatment frequency
**D3N**	2.4 % (2/82)	16.6 % (1/6)
**A75V**	6.1 % (5/82)	16.6 % (1/6)
**T79M**	6.1 % (5/82)	16.6 % (1/6)
**T94F**	0 %	16.6 % (1/6)
**S98G**	14.45 % (12/82)	50 % (3/6)
A30K	6.1 % (5/82)	33.3 % (2/6)
A62T	24.4 % (20/82)	50 % (3/6)
Y93H	0 %	66.6 % (4/6)

**Table 2.** Presence of substitutions in the samples from patients who relapsed therapy

Substitution				
Post-treatment sample number*	*A30K*	*A62T*	*Y93H*	*S98G*
P11	−	−	**+**	**+**
P45	−	−	**+**	−
P112	**+**	**+**	−	−
P175	−	**+**	**+**	**+**
P189	**+**	**+**	−	**+**
P192	−	−	**+**	−

*Numbers are identification codes from a biobank of HCV samples held at São Paulo State University.

#### *In vitro* replication phenotype of S98G

To evaluate the fitness impact of this substitution during viral genome replication, site-directed mutagenesis was performed to generate S98G derivatives of two different genotype 3 SGRs: S52 and DBN3a. The SGR-luc-S52 was described previously [6] and the DBN3a SGR was derived from the DBN3acc infectious clone [9] and has been described elsewhere [8]. Both SGRs contained a modified firefly luciferase reporter with an engineered low frequency of CpG/UpA dinucleotides; we [6] and others [10] have previously shown that this enhances the replication of GT3 SGRs through an as yet undefined mechanism.

**Fig. 2.**
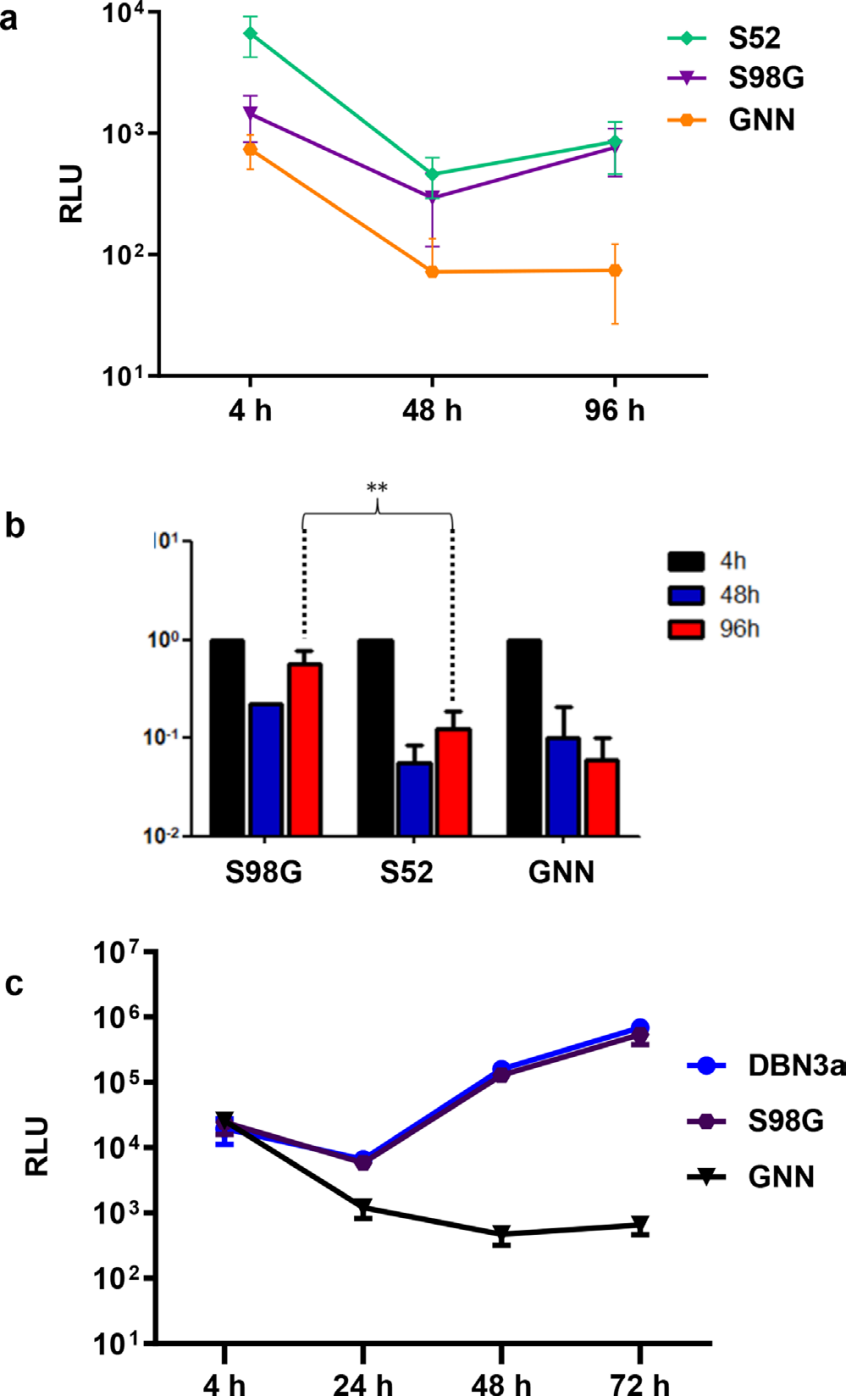
Replication phenotype of S98G. (a) S52 SGR RNAs (original, S98G and NS5B GNN) were electroporated into Huh7.5 cells and harvested at the indicated times for the luciferase assay. (b) S52 SGR replication values were normalized to the 4 h p.e. values (corresponding to translation of input RNA). *P*<0.01. (c) DBN3a SGR RNAs (original, S98G and NS5B GNN) were electroporated into Huh7.5 cells and harvested at the indicated times for luciferase assay.

Huh7.5 cells were electroporated with SGR-luc-S52 or SGR-luc-DBN3a, including the corresponding polymerase-inactive GNN mutants as negative controls, and luciferase levels were assayed in lysates harvested between 4–96 h p.e. to measure genome replication. For both SGRs, the S98G substitution was tolerated and allowed a similar replication level to the original. However, for S52 there was some evidence that S98G resulted in a modest enhancement of replication. As expected, S52 replicated poorly (Fig. 2a) and, although the input value (4 h) for S98G was significantly lower than the original, by 96 h both S98G and the original exhibited the same luciferase value, suggesting that S98G was able to replicate more efficiently. This was confirmed by the normalized data (normalized to the 4 h value) (Fig. 2b), showing that S98G did indeed replicate at a level that was statistically significantly higher than wild-type. In contrast, the DBN3a SGR replicated more efficiently than S52, and in this case all values for both S98G and the original were indistinguishable. We conclude that S98G can confer a modest increase in replicative fitness in HCV GT3a, although, in the context of an already efficiently replicating SGR, this enhancement was not observed.

### S98G resistance phenotype against DAAs

**Fig. 3.**
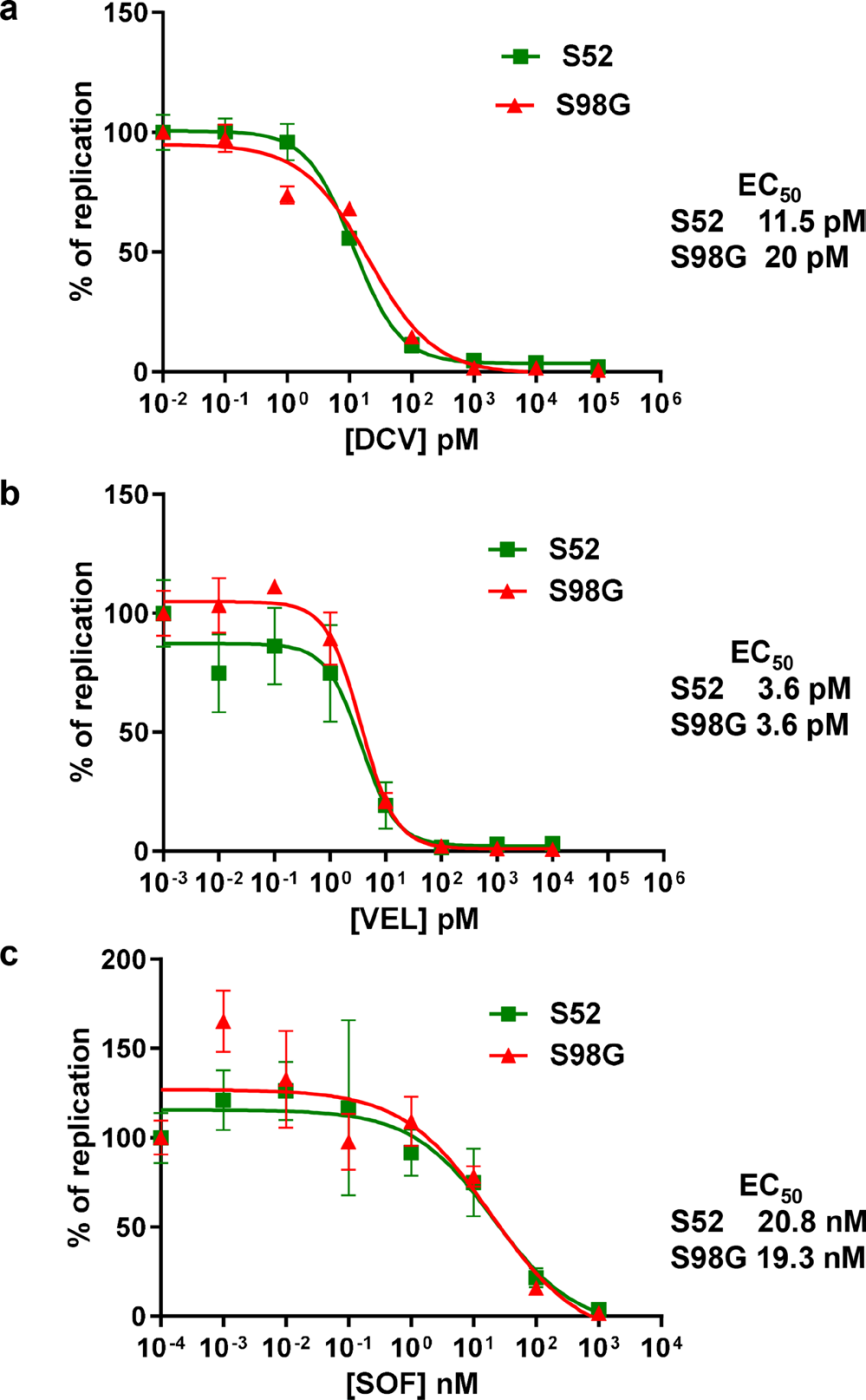
DAA resistance phenotype of S98G. S52 SGR RNAs (original or S98G) were electroporated into Huh7.5 cells, treated with DCV (a), VEL (b) or SOF (c) at the indicated concentrations for 96 h prior to harvest for luciferase assay. Values were normalized to the no-DAA controls. EC_50_ values for original and S98G are indicated on the right.

**Fig. 4.**
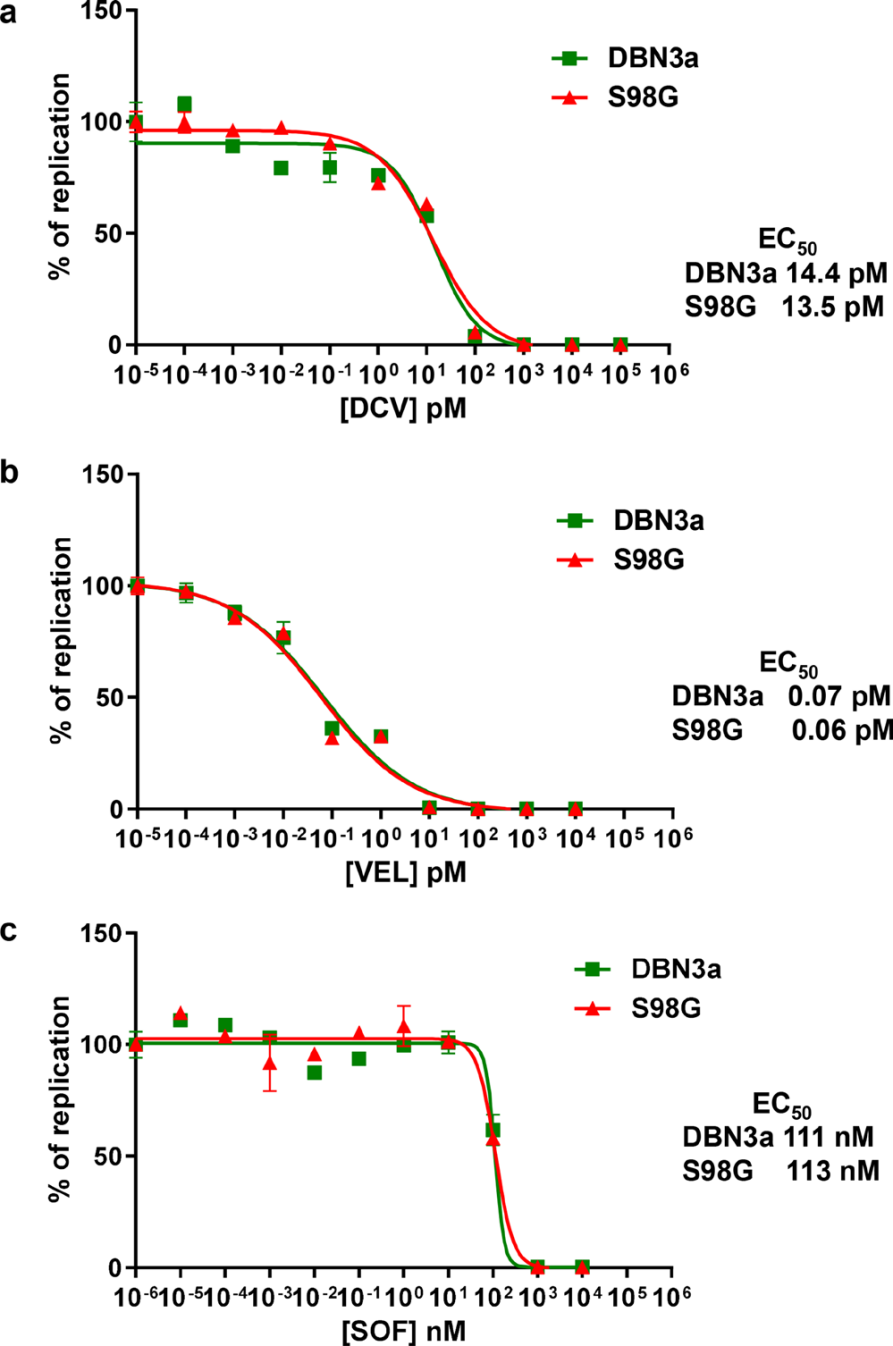
DAA resistance phenotype of S98G. DBN3a SGR RNAs (original or S98G) were electroporated into Huh7.5 cells, treated with DCV (a), VEL (b) or SOF (c) at the indicated concentrations for 72 h prior to harvest for luciferase assay. Values were normalized to the no-DAA controls. EC_50_ values for original and S98G are indicated on the right.

As S98G was associated with relapsing patients, we investigated the effect of this substitution on the response to two NS5A inhibitors: DCV (used in the clinical study) and velpatasvir (VEL) a third-generation DAA with potent pan-genotypic activity that was not available in Brazil whilst this patient cohort was being treated. Huh7.5 cells electroporated with SGRs were treated with DAAs at different concentrations to determine the effective concentration 50 % (EC_50_). As a control we also determined the EC_50_ for the NS5B inhibitor, SOF, a nucleoside analogue. For S52, the S98G substitution did have a modest effect on the DCV EC_50_ with an increase from 11.5 to 20 pM (Fig. 3a), although it is unlikely that this would contribute to clinical resistance. S98G had no effect on the VEL or SOF EC_50_ values for S52 (Fig. 3b, c). In contrast, for DBN3a (Fig. 4), the S98G substitution had no effect on the response to any of the DAAs tested.

### S98G phenotype in the presence of Y93H

This result was somewhat surprising and did not explain why S98G was strongly associated with the relapsing phenotype, as it appeared to confer very little selective advantage, either in the context of replicative fitness or DAA resistance. We reasoned that S98G might therefore confer some advantage when combined with other substitutions. To address this, we focused our attention on the well-characterized RAS, Y93H. We had previously demonstrated that, in our hands, Y93H resulted in a 71 000-fold increase in the DCV EC_50_ for the S52 SGR [6].

**Fig. 5.**
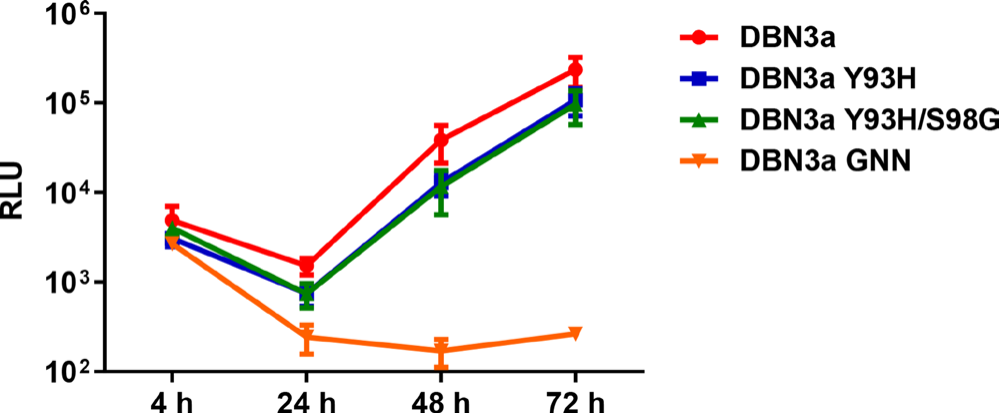
Replication phenotype of S98G in combination with Y93H. DBN3a SGR RNAs (original, Y93H, Y93H/S98G and NS5B GNN) were electroporated into Huh7.5 cells and harvested at the indicated times for luciferase assay.

Y93H was not detected in pre-treatment samples, with the proviso that analysis was by Sanger sequencing, so it is possible that Y93H was present as a minor variant below the frequency of detection. In contrast, it was detected in 66.6 % (4/6) of the post-treatment samples from the relapsed patients and, in two of these, this substitution occurred in conjunction with S98G. To test the potential phenotype of S98G in the context of Y93H, we generated SGR-luc-DBN3a derivatives with either Y93H alone, or the combination of Y93H/S98G. For this we focused on DBN3a as the replication levels were higher than S52, making it easier to identify any subtle differences in replication. Huh7.5 cells were electroporated with SGRs, including the polymerase-inactive GNN mutant as negative control, and harvested over a 72 h time period (Fig. 5). The data show that both Y93H and Y93H/S98G replicated to a similar level as the original. Although the luciferase values throughout for both were slightly lower than those for the original, this is most likely due to lower transfection efficiency, as evidenced by the 4 h time point. Importantly, the rate of replication for all three SGRs between 24–72 h was indistinguishable, clearly demonstrating that neither substitution impacted on replication fitness.

**Fig. 6.**
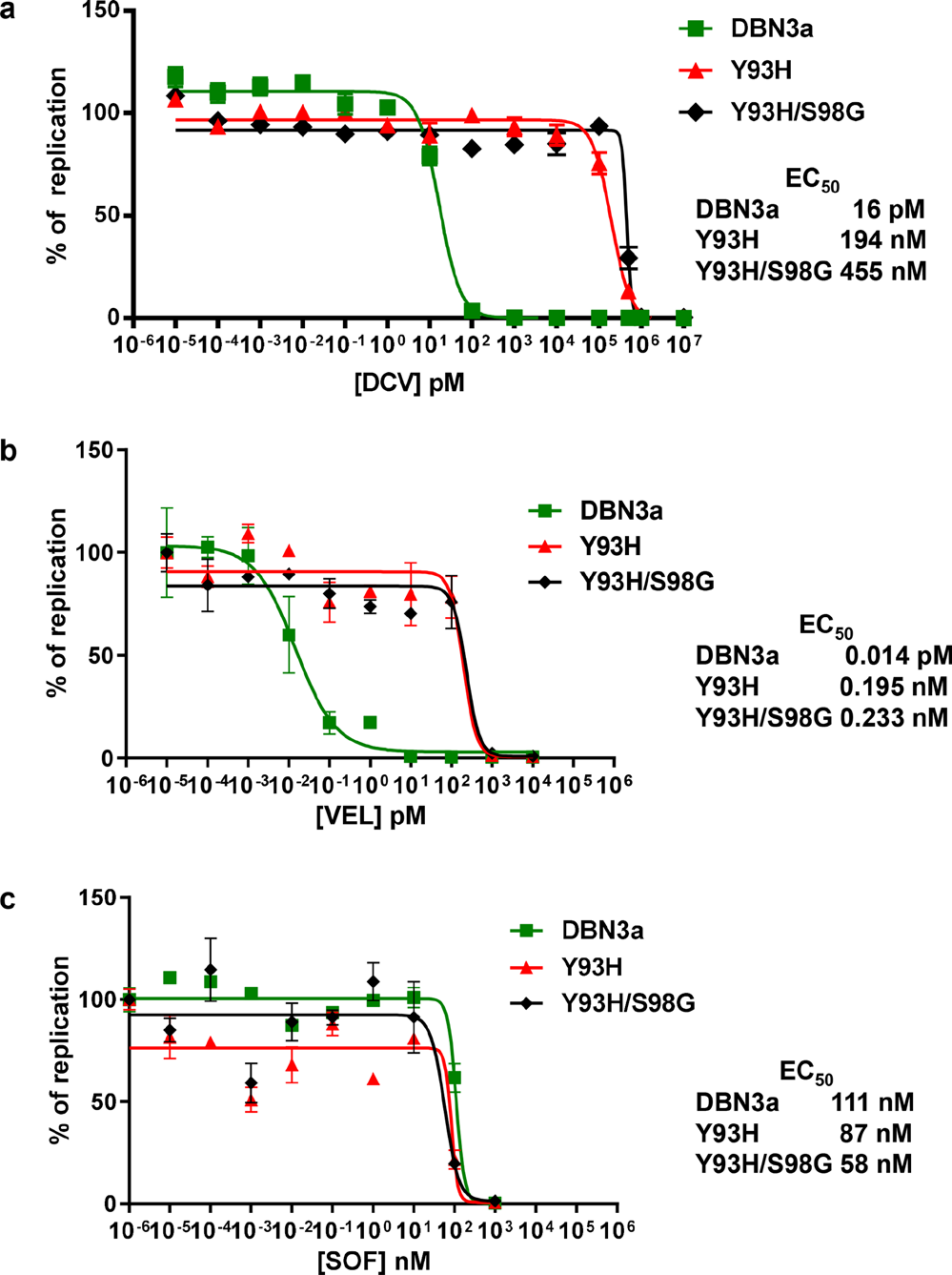
DAA resistance phenotype of S98G in combination with Y93H. DBN3a SGR RNAs (original, Y93H or Y93H/S98G) were electroporated into Huh7.5 cells, treated with DCV (a), VEL (b) or SOF (c) at the indicated concentrations for 72 h prior to harvest for luciferase assay. Values were normalized to the no DAA controls. EC_50_ values for original and S98G are indicated on the right.

We therefore evaluated the effect of Y93H and Y93H/S98G on the response of SGR-luc-DBN3a to DCV, VEL and SOF. As expected, Y93H resulted in a dramatic increase in the resistance of SGR-luc-DBN3a to DCV, increasing the EC_50_ from 16 pM to 194 nM (Fig. 6a). Reassuringly, the presence of S98G further increased the EC_50_ to 455 nM. The DBN3a SGR was highly sensitive to VEL, and again Y93H resulted in an increase of the EC_50_ from 0.014 pM to 0.195 nM (Fig. 6b). However, in this case S98G did not result in the acquisition of further resistance. Neither Y93H nor the combination of Y93H/S98G resulted in resistance to SOF compared to the original (Fig. 6c). Although the effect of S98G was modest, it did provide an explanation for the selection of S98G in samples from patients who relapsed following DCV treatment.

## Discussion

In this study we identify a novel substitution, S98G, in NS5A of GT3a-infected patients who relapsed following DAA treatment. The strict definition of an RAS is a substitution from the consensus that conferred reduced sensitivity of a virus to one or more antiviral drugs. The data presented here demonstrate that this is not the case for S98G and thus it cannot be specifically defined as an RAS. However, our data clearly show that in combination with the well-characterized RAS Y93H, it can enhance resistance to DCV.

The frequency of S98G was significantly higher in relapsed patients post-therapy compared to pre-treatment and we considered that it might be contributing directly to treatment failure. If this was the case, S98G would need to maintain replicative fitness and result in an increase in DCV EC_50_. To evaluate whether this was the case, we investigated the phenotype of the corresponding mutation in the context of two GT3a SGRs derived from the S52 [7] and DBN3a [9] infectious clones. This analysis revealed that S98G had no deleterious effect on the replicative capacity of either SGR; indeed, in the context of the poorly replicating S52 SGR, it exhibited a modest enhancement of replication (Fig. 2a, b). Thus, there would be no selective disadvantage of S98G, and S98G could persist in a viral population, as it had no replicative defect.

S98G alone had little or no effect on the EC_50_ for DCV in the context of either SGR (Figs 3 and 4). Although there was a modest increase in the S52 EC_50_ (from 11.5 to 20 pM), this would not be expected to have a clinically significant effect. The plasma concentration of DCV in the 24 h period following a single daily dose of 60 mg was between 270–2700 nM [11]. Although modelling analysis suggests that tissue concentrations are 10-fold lower [12], this would still give a concentration of between 27–270 nM in the liver. This did not explain the apparent selection of S98G in relapsed patients. We therefore considered that S98G might act additively, or synergistically, with other substitutions to further increase EC_50_ values. An obvious candidate was Y93H, which was also observed in the relapsed patient post-treatment samples. To test this, we analysed the phenotype of S98G when combined with Y93H in the context of the DBN3a SGR and compared this with either the original or Y93H. SGR-luc-DBN3a Y93H or Y93H/S98G replicated as well as the original (Fig. 5), indicating that the combination of these two substitutions did not adversely affect replicative capacity and could therefore persist in a viral population. Reassuringly, compared to Y93H, the combined Y93H/S98G SGR exhibited a further increase in DCV EC_50_, from 194 to 455 nM (Fig. 6). Although this is only a ~twofold increase, we believe that it is potentially clinically significant – if the maximal DCV concentration in the liver is 270 nM, then this level of DCV would be predicted to inhibit both the original and Y93H, but would be less effective against Y93H/S98G. Although by necessity speculative, this conclusion does provide some indirect evidence for a selective advantage of the Y93H/S98G combination during DCV therapy. In contrast, S98G had no effect on the EC_50_ for VEL – both Y93H and Y93H/S98G exhibited a similar EC_50_ for this DAA (Fig. 6). This has implications for the choice of DAA regime – patients with baseline Y93H/S98G RAS should be treated with a DAA combination that contains VEL, not DCV. Indeed, this is now the case in many countries, including Brazil, so the presence of S98G may have less clinical impact than previously expected. As the two substitutions are only 15 nucleotides apart, it should be relatively easy to identify linkage via either next-generation or conventional Sanger sequencing approaches. Interestingly, S98G was observed in a subset of the relapsed patients in the absence of Y93H (Table 1), suggesting that it might also contribute to resistance in cooperation with other previously defined RASs (e.g. A30K or A62T).

**Fig. 7.**
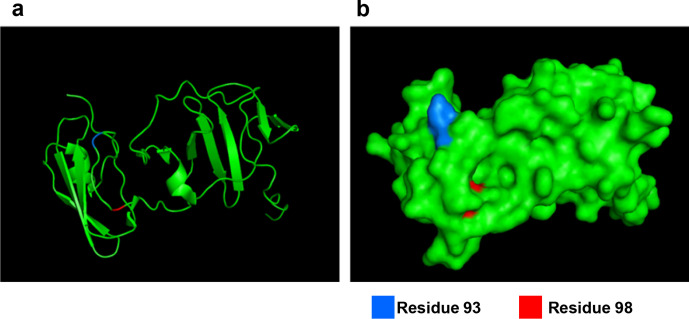
NS5A domain I monomer tertiary structure and surface representation, showing the localization of Y93 (in blue) and S98 (in red).

So, how might S98G cooperate with Y93H to drive high-level resistance to DCV? Even if not currently of clinical significance, could S98G provide some insight into the obscure mode of action of the NS5A-targeting DAAs? To address this we mapped the two substitutions onto the three-dimensional structure of domain I of genotype 1b NS5A [13, 14] (Fig. 7). Although close to each other in the primary amino acid sequence (Fig. 7a), the two residues are not proximal within the structure. In particular, residue 93 is surface-exposed, whereas residue 98 is partially buried (Fig. 7Bb). However, it is still plausible that both residues form key contact points for DCV binding, or, alternatively S98G might have an allosteric effect on the structure of NS5A. The other implication of the data is that the potential interaction of VEL with NS5A is subtly different than that of DCV and does not involve residue 98. VEL is clearly a more potent DAA in comparison to DCV, but is equally affected by Y93H (Fig. 6), and so it is likely that VEL contacts other residues within NS5A, perhaps residues that are more functionally constrained and therefore cannot mutate to confer resistance.

Our data are consistent with the notion that the resistance of HCV GT3 to NS5A-targeting DAAs is driven by multiple substitutions and varies with respect to the specific DAA being administered to the patient. Consideration must be given to baseline substitutions when deciding on clinical approaches. The identification of novel substitutions such as S98G may contribute to our understanding of the molecular mechanisms of, and resistance to, NS5A-targeted DAAs, and may also be a useful tool to investigate NS5A functions in the virus lifecycle. In this context, it will be intriguing to study the phenotype of S98G in the context of infectious virus, particularly in light of our previous observation that NS5A domain I plays a role in virus assembly [15]. Such experiments are planned in our laboratory for when work can recommence following the coronavirus disease 2019 (COVID-19) lockdown.
