# Corrigendum: A novel substitution in NS5A enhances resistance of hepatitis C virus genotype 3 to daclatasvir

**DOI:** 10.1099/jgv.0.001582

**Published:** 2021-03-31

**Authors:** Guilherme Rodrigues Fernandes Campos, Joseph Ward, Shucheng Chen, Cintia Bittar, João Paulo Vilela Rodrigues, Ana de Lourdes Candolo Martinelli, Fernanda Fernandes Souza, Leonardo Régis Leira Pereira, Paula Rahal, Mark Harris

**Affiliations:** ^1^​ São Paulo State University, Institute of Biosciences, Languages and Exact Sciences, São José do Rio Preto, São Paulo State 15054-000, Brazil; ^2^​ School of Molecular and Cellular Biology, Faculty of Biological Sciences, and Astbury Centre for Structural Molecular Biology, University of Leeds, Leeds, LS2 9JT, UK; ^3^​ University of São Paulo, Ribeirão Preto School of Medicine, Ribeirão Preto, SP 14049-900, Brazil; ^4^​ University of São Paulo, Ribeirão Preto Faculty of Pharmaceutical Sciences, Ribeirão Preto, SP 14040-903, Brazil

In the published version of this article, a grant number was omitted from the section ‘Funding Information’. The section should have read:

This work was funded by an MRC grant (MR/S001026/1) to M. H., while S. C. was funded by the China Scholarship Council. G. R. F. C., C. B. and P. R. were funded by FAPESP (grant numbers 2016/03807-0, 2017/22927-0 and 2018/04678-5).

The authors apologise for any inconvenience caused.

